# Adherence to Remote Prescribing Principles by Medical and Non‐Medical Prescribers; a Scoping Review

**DOI:** 10.1111/jan.70198

**Published:** 2025-09-08

**Authors:** Michelle Lewington, Ruth E. Paterson, Sharad Rayamajhi, Sonya Macvicar

**Affiliations:** ^1^ School of Health and Social Care Edinburgh Napier University Edinburgh UK

**Keywords:** e‐prescribing, remote consultation, remote prescribing, remote prescribing governance, safety, scoping review

## Abstract

**Aim:**

To examine the extent of adherence to high‐level principles in remote prescribing and investigate how medical and non‐medical prescribers comply with these principles.

**Design:**

Scoping Review.

**Data Sources:**

A systematic search of CINAHL, PubMed, Medline, the Cochrane Database of Systematic Reviews, the Web of Science, and the Ovid Emcare databases was performed. A grey literature search was conducted on relevant professional websites and Google Scholar. Literature was searched from January 2007 to March 2025.

**Review Methods:**

Research results were uploaded to Raayan for management and selection of evidence. Two reviewers independently scored and appraised papers using a structured data extraction form. The ‘United Kingdom High‐level Principles for Good Practice in Remote Consultations and Prescribing’ served as a coding framework for deductive manifest content analysis.

**Results:**

Searches identified 6870 studies. After screening the title and abstract, 54 full texts were reviewed, and 14 studies were identified for analysis. Adherence to high‐level principles was limited and inconsistent. Data categories were developed into 5 themes: (1) Patient privacy and vulnerability, (2) Adequate assessment, (3) Guidelines and evidence‐based prescribing, (4) Investigations and safety netting, and (5) Organisational safety and creating safe systems.

**Conclusion:**

This review provided insight into the challenges that medical prescribers face when adhering to governance principles during remote prescribing practice. However, no research about how non‐medical prescribers integrate remote prescribing governance into their practice was found.

**Impact:**

Remote prescribing has become firmly embedded within the current healthcare system and robust governance is required to safeguard patient outcomes. Further research exploring how non‐medical prescribers integrate the high‐level principles into practice will inform prescribing governance for this group.

**Patient or Public Contribution:**

No patient or public contribution was sought as the scoping review focused solely on the existing literature.

## 1 Introduction

The growing health complexity of the population has resulted in more health professionals with legislative authority prescribing medications, an increase in prescribing activity, and a shift from traditional in‐person consultation to remote assessment. Prescribing is now the most common therapeutic intervention in healthcare, with medicines‐related harm recognised as a global patient safety issue (World Health Organisation [WHO] 2019). Deslandes et al. (2022) reported an increase in prescribing activity during a 10‐year longitudinal study with the number of items prescribed by general practitioners and non‐medical prescribers growing from 417,000 in 2011 to 2.2 million in 2021. Similarly, in Scotland, a cross‐sectional study demonstrated a 125% increase in prescribing activity within primary care from 2013 to 2022, with a 400% increase in proton pump inhibitor prescribing during that period (MacVicar and Paterson 2023).

The UK remains the global leader in the breadth of non‐medical prescribing rights, while other countries have adopted more limited or collaborative models (Kroezen et al. 2011). A range of non‐medical healthcare professionals, such as nurses, midwives, allied health professionals, and pharmacists, can prescribe medicines as independent non‐medical prescribers (NMP). Whilst the scope of prescribing authority varies across these professions, they are responsible and accountable for health assessment, clinical decision making, and the prescribing outcome. In the context of remote prescribing, technological advancements have facilitated the practice; since 2008, a shift to remote prescribing for various chronic conditions has occurred (Crossley et al. 2011; Flodgren et al. 2015). The COVID‐19 pandemic resulted in a spike in demand, which has continued (Wong et al. 2021). However, some scholars, such as Robertson (2021) have questioned whether the inability to perform a detailed holistic patient assessment during remote consultation impacts the prescribing decision and raises the potential for medicines‐related harm.

### 1.1 Background

Remote prescribing is defined as a prescribing decision that is undertaken when the patient and practitioner are in different geographical locations (Broadhead 2020). It aims to provide timely access to expert advice and access to medicines, regardless of geographical location, and has been endorsed by medical, nursing and midwifery regulators (GMC & NMC). Following concerns raised by the Care Quality Commission (CQC), UK healthcare regulators published a joint statement committing to develop shared principles for evidenced‐based, safe and effective remote prescribing (CQC 2019; GMC 2019). Collaboration between 13 professional regulators identified best practices and outlined standards that all prescribers should meet prior to, and during, a remote prescribing consultation (GMC et al. 2019; HCPC 2019; NMC 2019). These standards are aligned to international best practice which includes requirements for governance at systems level, assessment of vulnerability, robust consultation prior to prescribing, verification of privacy, access to medical records, and effective safety netting and follow‐up after consultation (Medical Board of Australia 2023; Gullslett et al. 2024). Despite the similarities between global and UK standards, the authority to remotely prescribe varies between countries (Sharma et al. 2023). For example, in Thailand, remote prescribing is not permitted, and in India and Nepal, prescribing is restricted to certain categories of medication. In Australia, asynchronous remote consultation and prescribing via chatbots, social media, or discussion boards are not permitted, whereas in the USA, there are legal requirements that vary from state to state, with some permitting remote prescribing for all medications and others restricting prescribing for abortions and controlled substances (American Association for Family Practice (AAFP) 2024).

Remote prescribing has global reach and offers a flexible alternative to face‐to‐face consultations. However, the shift from in‐person consultation poses therapeutic and professional challenges relating to the assessment of privacy, dignity, and capacity to inform diagnosis and prescribing decision (Royal Pharmaceutical Society 2021).

A cohort study involving 45,997 consultations found that during remote consultations, patients were 23% more likely to be prescribed antibiotics compared to those seen in face‐to‐face consultations (Vestesson et al. 2023). Similarly, a multi‐method qualitative study examined 95 patient safety incidents that occurred during remote primary care encounters and found that, whilst adverse events were ‘extremely rare’, 15 cases of death and serious harm such as missed diagnosis, delayed referral and incorrect treatment were reported (Payne et al. 2023). The study highlighted that remote prescribing might interfere with communication, rapport building and identifying non‐verbal cues. This indicates that although remote prescribing increases access to medicines, it also carries the risk of harm to the service user associated with digital exclusion, inefficiency, technological failure and the potential to compromise the quality of the consultation (Payne et al. 2023). As a result, there is a need for a comprehensive understanding of its practical implementation and alignment of the intervention with international best practice, thereby informing the direction of future research and practice in this area. In response, ML, SR, RP and SM conducted a scoping review to explore existing literature on the topic.

## 2 The Review

### 2.1 Aim

The aim of the review was to describe global evidence on how remote prescribers ensure quality, safety and governance in their practice. The high‐level principles for good practice in remote consultation and prescribing were used as a comprehensive, internationally relevant framework.

### 2.2 Design

This scoping review was guided by the Joanna Briggs Institute's (JBI) approach (Pollock et al. 2021) to mapping existing literature, identifying key concepts and clarifying knowledge gaps. The process began with the development of research questions which were aligned with the overall objective of the review.

The review aimed to address the following research questions:

1. How do independent prescribers adhere to and implement quality, safety, and governance measures when undertaking remote prescribing practice?
2. What are the areas for future research and practice development?

The search strategy was underpinned by the Participants, Concept and Context (PCC) framework to maximise replication strength and rigour (Peters et al. 2020).

### 2.3 Search Methods

The next step involved an initial search of the CINAHL and PubMed databases to confirm keyword and medical subject headings. Secondly, additional synonyms were identified and applied during a systematic search of CINAHL, PubMed, Medline, the Cochrane Database of Systematic Reviews, the Web of Science, and Ovid Emcare. Finally, the reference lists of the included studies were reviewed for relevant publications, and a grey literature search was conducted using professional websites and Google Scholar. An example of search terms can be found in File S1. The search strategy underwent a Peer Review of Electronic Search Strategies (PRESS) to ensure its rigour and high quality (McGowen et al. 2016).

This scoping review protocol was registered with the Open Science Framework Registry (OSF) (see https://osi.io.auqi2/ Identifier DOI 10.17605/OSF.IO/4HXF7).

### 2.4 Eligibility Criteria

The inclusion criteria were: (1) any remote consultation resulting in prescribing being considered by either a doctor or NMP, (2) papers discussing how remote prescribing was implemented in clinical practice, (3) papers describing implementation in private health care settings, general practices, or any national health services in any country worldwide, (4) consultations delivered via telemedicine, e‐prescribing, e‐consultations, video consultations, or close‐to‐me consultations. Papers published from 2007 onwards. Only papers published in English were included. The eligibility criteria align with the PCC framework recommended by the JBI (Peters et al. 2020) (Table 1). The justification for the inclusion criteria was provided in File S2.

**TABLE 1:** Eligibility criteria based on PCC.

	Inclusion criteria	Exclusion criteria
Participants	Includes any type of remote consultation resulting in prescribing being considered by either a doctor or NMP	Health professionals who are not medical doctors or NMPs
Concept	Literature that describes quality, safety or governance structures implemented whilst undertaking remote consultations and prescribing Literature dated from 2007‐date	Literature that does not refer to how quality, safety or governance Literature that does not discuss remote consultations and prescribing quality, safety or governance
Context	Private health care practices, general practices and national health care services. Evidence acquired from any country worldwide Telemedicine, e‐prescribing, e‐consultations, video consultations, close‐to‐me consultations	Face‐to‐face consultations

### 2.5 Study Selection

Literature was searched from January 2007 to March 2025. Initial searches were imported into Rayyan and reviewed within the Mendeley web library, and independently screened by the first and second author, first by title and abstract and then by full text. All relevant abstracts were screened to determine their eligibility for full‐text review. A subset of 100 papers from the database was independently reviewed by ML and SR to reach a consensus on which papers met the inclusion criteria and which were to be excluded. Any disagreements were discussed with RP and SM to reach a consensus. Consistent with scoping review and content analysis methodology, quality appraisal was not carried out (Pollock et al. 2021).

### 2.6 Data Extraction

Data was extracted using the data extraction table guided by the JBI guidance and aligned with the review questions (Peters et al. 2020). Data concerning safety and governance were identified and subsequently organised using the UK high‐level principles for good practice in remote consultations and prescribing (NMC 2019; HCPC 2019) as a coding framework. This framework was selected due to the absence of any other appropriate framework, the clarity of the principles, comprehensive overview, and alignment with global standards of telehealth and remote prescribing.

### 2.7 Data Analysis

**FIGURE 1:**
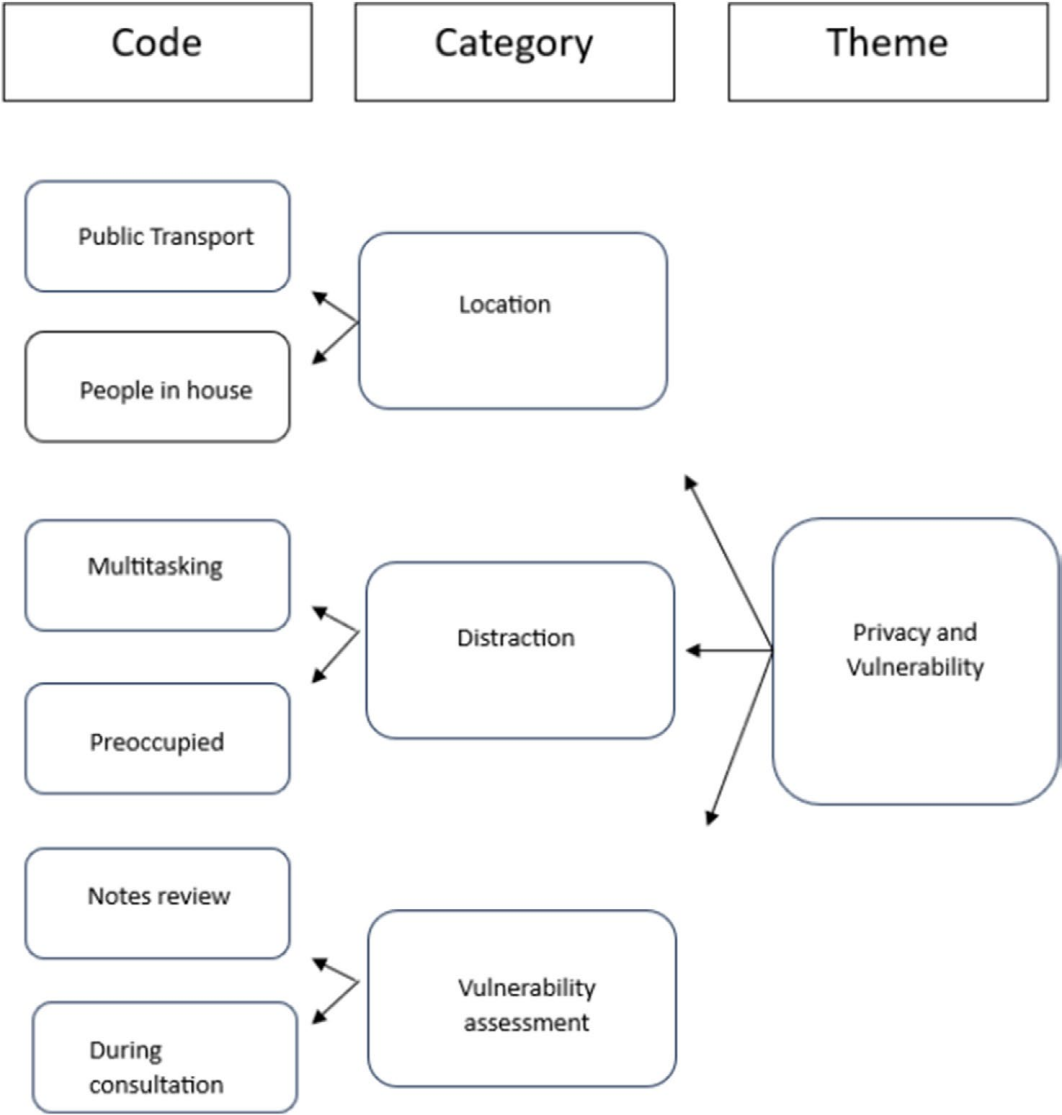
Example of how codes were developed into themes.

The review used deductive manifest content analysis to analyse the included studies (Schreier 2012). Manifest content analysis is a systematic method used to analyse surface‐level data. The first step in this methodology involved formulating the research questions and identifying data for analysis (Krippendorff 2018). Next, a theoretical coding framework was developed by the research team, using the high‐level principles for good practice in remote consultations and prescribing framework (NMC 2019; HCPC 2019) thus enabling data to be coded into categories (Table 2). The data was then analysed by the first author and verified by the fourth author. Disagreements were moderated by the third author and consensus was reached. Finally, findings were interpreted in the context of the research questions and categories themed through author consensus (see Figure 1) (Kleinheksel et al. 2020).

**TABLE 2:** Coding framework.

Code	Code description	Examples of code
Safety/Escalation/Identity checks	Ensure safety, identity verification and escalation of concerns	Use of end‐to‐end encryption Identity confirmation before consultation Privacy for patient and practitioner Escalation process for reporting inadequate technology or interruptions
Vulnerability	Identifying and managing vulnerable patients	Reviewing notes/alerts preconsultation Identifying vulnerability through age, behaviour, history, or medication requests Escalation to face‐to‐face consultation or specialist referral
Introductions/Scene setting	Introducing oneself and ensuring the patient understands the remote consultation	Providing name and role clearly Confirming patient's understanding of remote consultation Allowing time for patient questions and addressing concerns
Expectations management	Setting realistic expectations about remote prescribing and sharing of information	Explanation of limitations of remote consultation Gaining consent to share information with other services if needed
Consent/Mental capacity	Ensuring informed consent and assessing mental capacity	System alerts about mental capacity Confirming patient understanding and agreement Undertaking mental capacity assessment when necessary
Clinical assessment/Records/Results	Ensuring adequate information is available for safe prescribing decisions	Patient reported reading (Blood pressure/glucose levels/oxygen saturation, etc.) Reviewing of pictures (e.g., wounds, rashes) Considering all available investigation results Referral for further investigations
Information provision	Providing patients with clear, understandable treatment options	Consultation overviews Asking patient follow‐up questions to ensure understanding Advising on treatment options, including declining treatment
Safety netting and shared care/referral	Arrangements for aftercare/tests/monitoring/treatment. Sharing information with other healthcare providers	Advising of referrals to specialists or other healthcare providers Referring for follow‐up and tests Providing safety net advice for monitoring symptoms or outcomes
Note taking	Documentation of consultation	Recording consultations using secure systems Providing narrative explanations for decisions
Training (general) and remote context	Receives relevant training to undertake a role in remote prescribing practice	Participation in any remote prescribing training Practice specific training. Undertaking continuous professional development
Working in safe systems	Ensuring safe environments, equipment, and governance structures	Formulary restrictions Prescribing restrictions relevant to role Provision of policies/guidance Provision of training specific to remote prescribing
Recognition of limitations of remote prescribing	Limitations to the scope of practice and formulary	Acknowledging limitations to practice scope and medication range Referring cases for face‐to‐face consultations

## 3 Results

### 3.1 Search Outcomes

**FIGURE 2:**
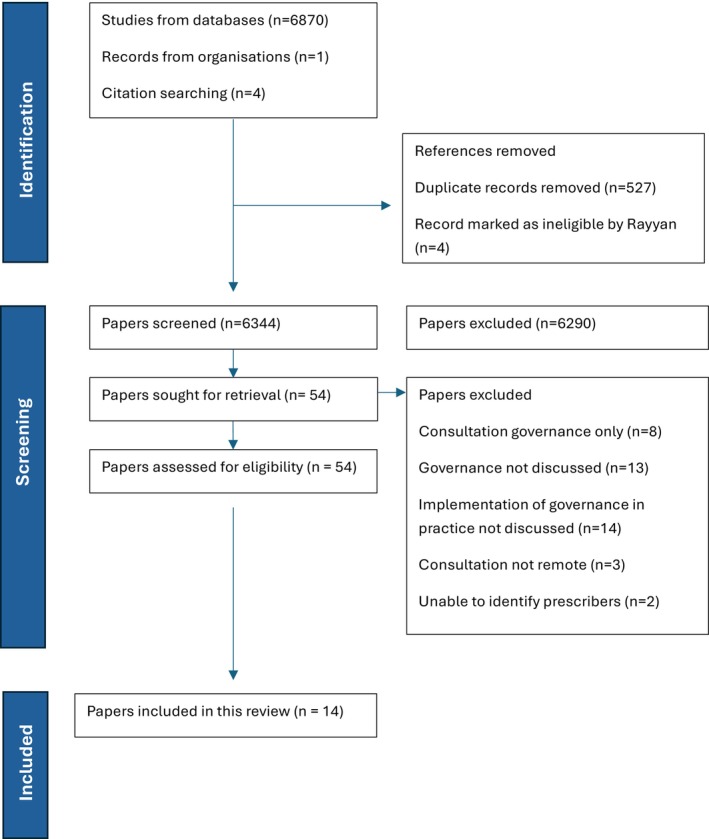
‘PRISMA’ flowchart.

The database search yielded 6870 articles. Following the removal of 523 duplicates and four ineligible records, 6343 titles and abstracts were screened. This resulted in 54 articles being selected for full‐text review. Of these, 40 were excluded for the following reasons: eight did not include any reference to prescribing, 27 did not describe the implementation of safety or governance measures during consultations or prescribing, and in the remaining six articles, the individual responsible for the consultation or the setting of the consultation was not clearly identified. This process resulted in 14 articles meeting the inclusion criteria (Figure 2). The included papers were published between October 2020 and March 2025.

### 3.2 Study Characteristics

The general characteristics of each included study are shown in Table 3. Of the 14 papers, three were conducted in North America, three in the UK, five in Europe, two in Australia and one in Oman. Nine papers were qualitative in design (Björndell and Premberg 2021; Gomez et al. 2021; Hasani et al. 2020; Hedden et al. 2023; Nguyen et al. 2024; Norberg et al. 2025; Rosen et al. 2022; Uscher‐Pines et al. 2020; Verhoeven et al. 2020), two were quantitative (Johnsen et al. 2021; Pogorzelska et al. 2023), and three employed a mixed methods design (Mahmood et al. 2024; Murphy et al. 2021; White et al. 2024). A total of 1936 practitioners were included in the studies (range 1–1237): 97.5% (*n* = 1887) identified as medical practitioners; 1.7% (*n* = 33) as psychiatrists; 0.6% (*n* = 13) as nurses and 0.2% (*n* = 3) as midwives.

**TABLE 3:** Included studies.

Study	Aims	Demographics	Methodology, data collection & analysis	Finding summaries supporting safety and governance
Björndell and Premberg (2021) Sweden	To describe physicians' experience of video consultation with new patients visiting a publicly owned virtual care clinic	1 Publicly owned virtual care clinic 10 primary care physicians Video consultations	Qualitative Semi‐structured interviews Systematic text condensation	*Implementation of governance*: Accurate assessment, patient self‐examination and patient information refer to face‐to‐face assessment *Safety and governance assessment*: Cognitive assessment, ensuring privacy and technology stability *Organisational and self‐governance adherence*: Patient selection and own limitations
Gomez et al. (2021) Southern California (USA)	Physicians' perspective on the rapid adoption of telemedicine during the COVID‐19 pandemic	15 family physicians Southern California academic health system Telephone and Video Consultations	Qualitative—semi‐structured Interviews Thematic analysis	*Implementation of governance*: Acknowledges insufficient assessments and escalation to face‐to‐face appointments, and follow‐up arrangements *Safety and governance assessment*: Communication assessment *Organisational And self‐governance adherence*: Limitations to medications and patient selection
Hasani et al. (2020) Muscat, Oman	Explores family physicians' perceptions of the use of telephone consultation concerning the implementation process, challenges and limitations	22 family physicians Various locations Telephone Consultations	Qualitative—interviews Interpretive Phenomenology	*Implementation of governance*: Refer for face‐to‐face assessment, arrange follow‐up, and note taking *Safety and governance assessment*: Safety and **v**ulnerability assessment, and ability to use technology *Organisation and self‐governance adherence*: Provision of and adherence to guidelines, staff training, patient group restrictions and staff experience
Hedden et al. (2023) Canada	Exploring Canadian family physicians' perspective of the rapid move to virtual care during the COVID‐19 pandemic	68 family physicians 4 regions in Canada Telephone and Video Consultations	Qualitative—semi‐structured interviews Thematic analysis	*Implementation of governance*: Safety netting, review photos, adequate assessment, referral for face‐to‐face, and note taking *Safety and governance assessment*: Technology and technical support, evaluation of medical records, privacy and confidentiality *Organisation and self‐governance adherence*: Training, building protocols and an organisational agreed triage system
Johnsen et al. (2021) Norway	Documenting GPs' experiences with large‐scale uptake of video consultations during the COVID‐19 pandemic.	1237 GPs in Norway Video Consultations	Quantitative surveys Descriptive statistics, diagrams and chi‐square tests	*Implementation of governance*: adequate assessment needed. Safety netting, referrals and follow‐up, advice and guidance *Organisation and self‐governance adherence*: Suitability for remote prescribing and caution with prescribing antibiotics
Mahmood et al. (2024) Leicestershire (UK)	An online survey exploring whether GPS and general practitioners' speciality trainees (GPSTS) had sufficient training to undertake telephone consultations and the impact that telephone consultations have on their clinical practice	85 GPs 48 GPSTs Telephone	Qualitative questionnaires Descriptive statistics and chi‐square	*Implementation of governance*: Adequate clinical assessment *Safety and governance assessment*: Communication and privacy assessment *Organisation and self‐governance adherence*: Training in remote prescribing, practice supervision and privacy assessment
Murphy et al. (2021) Bristol (UK)	To investigate the rapid implementation of remote consulting and explore its impact over the initial months of the COVID‐19 pandemic	21 GPs 11 practice managers 9 Senior nurses 1 Advanced nurse practitioner Telephone and Video Consultations	Mixed methods Quantitative – longitudinal analysis of consultations—descriptive statistics Qualitative—Longitudinal interviews with a purposive sample of practice staff	*Implementation of governance*: Adequate clinical assessment, photographic assessments, safety netting, referrals and information provision *Safety and governance assessment*: risk to patients and communication barriers *Organisation and self‐governance adherence*: Provision of technology and team meetings
Nguyen et al. 2024 Sydney Australia	Exploration of interactional components of telemedicine	15 GP's 9 patients Telephone and Video Consultations	Qualitative semi‐structured interview Thematic analysis	*Implementation of governance*: Adequate patient assessment, checking patient's understanding, safety netting, providing patients with information, taking notes, sharing notes and ordering tests *Safety and governance assessment*: Privacy assessment *Organisation and self‐governance adherence*: Training in remote prescribing
Norberg et al. (2025) Norway	GP perspective on how communication has been affected moving to remote consultations	18 GP's Telephone, video and text based	Qualitative focus group interviews Thematic analysis	*Implementation of governance*: Adequate patient assessment, reviewing photos and videos, and escalation to face‐to‐face *Safety and governance assessment*: Privacy and capacity assessment
Pogorzelska et al. (2023) Poland	Assessment of benefits and challenges of Telemedicine during the COVID‐19 pandemic	8 Primary care clinics 30 participants (11 family medicine physicians, 13 family medicine resident psychiatrists, 3 nurses and 3 midwives) Telephone and Video Consultations	Quantitative—Semi‐structured interviews Analysis of interview content	*Implementation of governance*: Safety netting, adequate assessment, self‐examination, review of photos and documentation *Safety and governance assessment*: Assessment of medical records, assessment of vulnerability, and assessment of technology *Organisation and self‐governance adherence*: Availability of remote prescribing and use of guidelines
Rosen et al. (2022) United Kingdom	To develop an empirically based and theory‐informed taxonomy of risks associated with remote consultations.	176 clinicians and 43 patients Telephone, Video, text or email Consultations	Qualitative sub‐study (3 large multi‐site, mixed methods studies) Semi‐structured interviews and focus groups–thematic analysis	*Implementation of governance*: Safety netting, self‐examination, complete medical history, patient safety assessment, ‘red flag’ safety netting. *Safety and governance assessment*: Assessment of medical records, vulnerability, and technology *Organisation and self‐governance adherence*: Availability of remote prescribing
Uscher‐Pines et al. (2020) USA	To gain an understanding of the dramatic change in the delivery of mental health care, including the mode of telemedicine that psychiatrists used.	20 outpatient psychiatrists Five US states Telephone and Video Consultations	Qualitative Semi‐structured interviews Inductive and deductive approaches and matrix analysis	*Implementation of governance*: Safety netting and self‐monitoring *Safety and Governance Assessment*: Privacy and vulnerable assessment *Organisation and self‐governance adherence*: Availability of remote consultation, safety meeting and vulnerability identifiers on the system
Verhoeven et al. (2020) Belgium	To gain insight into the consequences of the COVID‐19 outbreak on the core competencies of general practice as they moved to telemedicine.	132 GPs in Flanders Telephone and Video Consultations	Qualitative Semi‐structured interviews Inductive framework analysis approach	*Implementation of governance*: Safety netting, referrals, questioning approach and self‐examination *Safety and governance assessment*: Safety meetings *Organisation and self‐governance adherence*: Availability of remote prescribing and peer support
White et al. (2024) Sidney Australia	The research objective is to produce evidence‐based resources to support the implementation of person‐centred communication when undertaking telehealth	15 GPs 9 patients Telephone and video consultations	Mixed methods Qualitative—video/audio analysed with sociolinguistic and discourse analysis Quantitative—surveys analysed using descriptive statistics	*Implementation of governance*: Information provision, checking understanding, alternatives to medication, safety netting, ordering tests and taking notes. *Safety and governance assessment*: Note and result checks, making records of RP, privacy assessment *Organisation and self‐governance adherence*: Using guidelines

The papers reported a variety of remote prescribing approaches. A single modality, namely video consultation, was used by Björndell and Premberg (2021) and Johnsen et al. (2021), whilst telephone consultation was adopted by Hasani et al. (2020) and Mahmood et al. (2024). The others used a combination of email, text message, telephone, and video consultation (Rosen et al. 2022; Gomez et al. 2021; Hedden et al. 2023; Murphy et al. 2021; Nguyen et al. 2024; Norberg et al. 2025; Pogorzelska et al. 2023; Uscher‐Pines et al. 2020; Verhoeven et al. 2020; White et al. 2024).

### 3.3 Alignment of Literature With Quality, Safety and Governance Measures

Each of the papers was scrutinised to assess how closely it aligned with the high‐level principles (GMC 2019; HCPC 2019; NMC 2019) (Table 4). The wording used in the table has been taken directly from the high‐level principles to ensure consistency. The publication by Pogorzelska et al. (2023) demonstrated the highest level of alignment, meeting eight out of the 12 principles. In contrast, Mahmood et al.'s (2024) study showed the lowest degree of alignment, meeting only two. The majority of studies (*n* = 6) were aligned with five of the identified principles (Björndell and Premberg 2021; Hedden et al. 2023; Johnsen et al. 2021; Murphy et al. 2021; Norberg et al. 2025; Rosen et al. 2022).

**TABLE 4:** Alignment to high‐level principles.

High level principles	Björndell and Premberg (2021) Sweden	Gomez et al. (2021) Southern California (USA)	Hasani et al. (2020) Muscat, Oman	Hedden et al. (2023) Canada	Johnsen et al. (2021) Norway	Mahmood et al. (2024) Leicestershire (UK)	Murphy et al. (2021) Bristol (UK)	Nguyen et al. (2024) Australia	Norberg et al. (2025) Norway	Pogorzelska et al. (2023) Poland	Rosen et al. (2022) United Kingdom	Uscher‐Pines et al. (2020) USA	Verhoeven et al. (2020) Belgium	White et al. (2024) Australia
Patient safety is a priority	✓	✓	✓	✓			✓	✓	✓	✓	✓	✓		✓
Identify vulnerable patients	✓		✓						✓	✓	✓	✓	✓	
Introductions to patients														
Explain boundaries of practice														
Obtain consent and follow relevant capacity assessment and laws									✓					
Adequate clinical assessment	✓	✓		✓	✓		✓	✓	✓	✓	✓	✓	✓	
Explore options and information provision	✓				✓		✓	✓		✓	✓			✓
Aftercare, referral and interagency communication		✓			✓	✓		✓	✓	✓	✓		✓	✓
Documentation			✓					✓		✓				✓
Training support and CPD			✓	✓		✓	✓	✓					✓	✓
Working in safe systems			✓	✓	✓		✓			✓			✓	✓
Recognising limitations of remote prescribing practice	✓	✓	✓	✓	✓			✓		✓			✓	✓

The most frequently aligned principles, discussed in 11 out of 14 publications, were ‘adequate clinical assessment’ (Björndell and Premberg 2021; Gomez et al. 2021; Hedden et al. 2023; Johnsen et al. 2021; Murphy et al. 2021; Nguyen et al. 2024; Norberg et al. 2025; Pogorzelska et al. 2023; Rosen et al. 2022; Uscher‐Pines et al. 2020; Verhoeven et al. 2020) and ‘patient safety’ (Björndell and Premberg 2021; Gomez et al. 2021; Hasani et al. 2020; Hedden et al. 2023; Murphy et al. 2021; Nguyen et al. 2024; Norberg et al. 2025; Pogorzelska et al. 2023; Rosen et al. 2022; Uscher‐Pines et al. 2020; White et al. 2024). In contrast, none of the publications discussed patient introductions or boundary setting. Just over half of the included studies (*n* = 9) addressed principles related to aftercare, referral processes, and interagency communication (Gomez et al. 2021; Johnsen et al. 2021; Mahmood et al. 2024; Nguyen et al. 2024; Norberg et al. 2025; Pogorzelska et al. 2023; Rosen et al. 2022; Uscher‐Pines et al. 2020; Verhoeven et al. 2020).

Five main governance and safety themes emerged from the papers: (1) Patient privacy and vulnerability (2) Adequate assessment (3) Guidelines and evidence‐based prescribing (4) Investigations and safety netting; and (5) Organisational safety and creating safe systems. Table 5 describes each theme.

**TABLE 5:** Description of themes.

Theme	Description
Patient privacy and vulnerability	How practitioners ensure patients' privacy, dignity, and protection of information. Maintaining patient confidentiality and addressing issues related to patients' vulnerability
Adequate assessment	How practitioners undertake accurate patient assessments. Ensuring comprehensive patient history and adequate physical examinations
Guidance and evidence‐based prescribing	How practitioners arrive at a prescribing decision
	Choosing appropriate medications, understanding dosages, and monitoring potential side effects while considering patient specific factors
Investigations and safety netting	How practitioners address the need for proper diagnostic investigations and establishing patient safety netting. Ensure follow‐up care, accurately interpret test results, and manage potential risks or complications
Organisational safety and creating safe systems	Highlights the governance available within healthcare settings to provide a safe system. The availability of and adherence to policies, collaborating with healthcare teams, and creating a safe environment to prevent errors and enhance patient care

#### 3.3.1 Patient Privacy and Vulnerability

Maintaining patient privacy during remote prescribing and consultation was mentioned in eight studies (Björndell and Premberg 2021; Hasani et al. 2020; Hedden et al. 2023; Nguyen et al. 2024; Norberg et al. 2025; Pogorzelska et al. 2023; Uscher‐Pines et al. 2020; White et al. 2024). It was reported that the quality of remote consultation was compromised when patients were outside the home, such as using public spaces or travelling on public transport, or when they took calls whilst at work (Norberg et al. 2025; Pogorzelska et al. 2023; Uscher‐Pines et al. 2020). Meanwhile, Björndell and Premberg (2021) and White et al. (2024) expressed concerns about privacy in a person's home where family members may be able to overhear.

Vulnerability assessments took place before consultation by reviewing patient records to identify cognitive impairment, language barriers, elderly patients, or known social issues. These took the form of either a relative risk assessment in the context of pre‐consultation triage or a pre‐consultation questionnaire via text and family involvement during consultation (Pogorzelska et al. 2023; Uscher‐Pines et al. 2020; Verhoeven et al. 2020; Gomez et al. 2021; Hasani et al. 2020).

The evaluation of mental capacity and the acquisition of informed consent were addressed in some studies (Björndell and Premberg 2021; Uscher‐Pines et al. 2020), with Norberg et al. (2025) noting the use of a mental health diagnostic scale during mental health assessment. Hasani et al. (2020) reported escalating the consultation to a face‐to‐face assessment if the patient was deemed to lack capacity.

#### 3.3.2 Adequate Assessment

Several papers stressed the need for an adequate patient assessment when prescribing remotely (Björndell and Premberg 2021; Gomez et al. 2021; Hedden et al. 2023; Johnsen et al. 2021; Murphy et al. 2021; Nguyen et al. 2024; Norberg et al. 2025; Pogorzelska et al. 2023; Rosen et al. 2022; Uscher‐Pines et al. 2020; Verhoeven et al. 2020). In some cases, assessment commenced pre‐consultation by reviewing patients' medical records and this prior familiarity was deemed to facilitate the remote assessment process (Gomez et al. 2021; Hedden et al. 2023; Johnsen et al. 2021). Notably, Björndell and Premberg (2021) observed that patients were required to be registered with their healthcare system to enable medical records to be reviewed before remote consultations could take place. However, Pogorzelska et al. (2023) noted in their study that physicians expressed concerns about the lack of availability of a full medical or surgical history affecting adequate assessment.

The importance of obtaining a comprehensive medical history to inform clinical decision‐making was a concern for many of the practitioners included in this review (Björndell and Premberg 2021; Gomez et al. 2021; Pogorzelska et al. 2023; Murphy et al. 2021; Nguyen et al. 2024; Norberg et al. 2025; Pogorzelska et al. 2023; Rosen et al. 2022; Verhoeven et al. 2020). Pogorzelska et al. (2023) and Rosen et al. (2022) highlighted that thorough history‐taking can often reduce the need for a full physical examination. However, Gomez et al. (2021) cautioned that significant barriers—such as language difficulties and technological limitations—can impair a prescriber's ability to conduct a complete assessment. Similarly, Murphy et al. (2021) warned that an inadequate history may result in unsafe prescribing decisions.

Alternatives to physical assessment during remote consultations included self‐examination (Björndell and Premberg 2021; Verhoeven et al. 2020), physical movement (Uscher‐Pines et al. 2020), photographs (Björndell and Premberg 2021; Hedden et al. 2023; Murphy et al. 2021; Norberg et al. 2025; Pogorzelska et al. 2023), or a combination of these. Self‐monitoring was helpful when managing chronic disease such as diabetes or hypertension (Pogorzelska et al. 2023; Uscher‐Pines et al. 2020; Verhoeven et al. 2020) and photographs were found to be particularly important during wound assessment (Norberg et al. 2025). Rosen et al. (2022) reported enlisting the help of family members in performing physical examinations during video consultations.

#### 3.3.3 Guidelines and Evidence‐Based Prescribing

Effective guidance and guidelines were considered pivotal for remote prescribing practice, as they provided a robust framework for decision‐making (Björndell and Premberg 2021; White et al. 2024). Björndell and Premberg (2021) utilised established clinical frameworks such as the ‘Centor criteria’ and rapid test results to aid their prescribing decisions for streptococcal tonsillitis, while White et al. (2024) reported using clinical guides to support their decision‐making during the prescribing consultation.

Björndell and Premberg (2021) and Gomez et al. (2021) highlighted the significance of adhering to local and national prescribing guidance, practitioners remaining vigilant about controlled drug prescribing and the rational use of medicine. Three studies identified the importance of balancing the risk between improving health and avoiding harm through overprescribing (Gomez et al. 2021; Murphy et al. 2021; Rosen et al. 2022), with participants expressing increased confidence in declining medications not included in their remote prescribing formulary.

#### 3.3.4 Investigations and Safety Netting

Verhoeven et al. (2020) reported the use of safety netting in remote prescribing practice by making onward referrals to occupational physicians or physical care units. Arranging aftercare or other investigations following remote consultation was reported in a further nine papers (Björndell and Premberg 2021; Gomez et al. 2021; Johnsen et al. 2021; Mahmood et al. 2024; Nguyen et al. 2024; Norberg et al. 2025; Pogorzelska et al. 2023; Rosen et al. 2022; White et al. 2024). This included referring patients to acute healthcare settings (Johnsen et al. 2021; Rosen et al. 2022; Verhoeven et al. 2020) or admitting them directly to hospital if their condition was unstable (Rosen et al. 2022). Murphy et al. (2021) reported that consultations were followed up with a text message providing further information.

The need to progress to a face‐to‐face consultation for patients with red‐flag symptoms, particularly in cases of newborns or suspected cases of cancer, was emphasised (Björndell and Premberg 2021; Hasani et al. 2020; Murphy et al. 2021; Pogorzelska et al. 2023; White et al. 2024), including the necessity of undertaking home visits for some children and deteriorating or bedridden patients (Rosen et al. 2022; Pogorzelska et al. 2023).

Hasani et al. (2020) reported adhering to specific guidelines for documenting telephone consultations as part of safety‐netting. Concurrently, documentation of remote consultations was deemed essential from a medical‐legal perspective (Hasani et al. 2020; Hedden et al. 2023; Pogorzelska et al. 2023; Rosen et al. 2022; Uscher‐Pines et al. 2020).

#### 3.3.5 Organisational Safety and Creating Safe Systems

Creating safe systems involved setting up the organisational framework required to enable efficient remote prescribing practices and organising the provision of training specific to remote prescribing practice (Björndell and Premberg 2021; Gomez et al. 2021; Hasani et al. 2020; Hedden et al. 2023; Johnsen et al. 2021; Murphy et al. 2021; Pogorzelska et al. 2023).

The availability of technology and technological support was identified as a prerequisite for conducting remote consultations (Björndell and Premberg 2021; Hasani et al. 2020). This included the roll‐out of e‐prescribing systems and using integrated photos and video links as key features of remote assessment (Murphy et al. 2021). Information technology support was accessed via a government virtual helpdesk (Hedden et al. 2023), while Rosen et al. (2022) attributed the successful implementation of remote consultation to digital maturity within their organisation. In addition, staff training was advocated to ensure that clinicians were adequately prepared to engage in remote assessment (Gomez et al. 2021) and to mitigate potential risks (Hedden et al. 2023), while in one study it was found that only experienced or senior practitioners conducted remote consultations (Hasani et al. 2020). Verhoeven et al. (2020) reported the use of an online peer support group whose members supported each other in responding to clinical governance queries.

The importance of training specific to remote prescribing practice was highlighted in the study by Murphy et al. (2021). However, Hasani et al. (2020); Hedden et al. (2023); Nguyen et al. (2024); and Mahmood et al. (2024) all reported practitioners having either limited or no training specific to remote prescribing practice. Nguyen et al. (2024) reported that when training was given, it focused on telemedicine technology and not on supporting practitioners to make safe prescribing decisions in the remote setting. Mahmood et al. (2024) found that a lack of training directly affected the practitioners' confidence in this setting.

## 4 Discussion

As far as ML, SR, RP and SM are aware, this is the first scoping review to explore alignment with, and the practical application of, governance principles in remote consultation and prescribing. The studies reviewed demonstrate innovative approaches to safe, effective remote prescribing, highlighting the importance of person‐centred assessment to optimise remote consultations, and showing how it provides timely access to medicines for vulnerable or hard to reach populations. However, only three studies were conducted in the UK with the focus on medical practitioners rather than nurses, midwives, allied health, or pharmacist prescribers. Consequently, insight into how the high‐level principles are applied in practice in the UK and by non‐medical prescribers is limited. Further research in the UK on remote prescribing with non‐medical prescribers to explore this perspective is essential.

Key interventions in remote prescribing such as verifying identity, protecting privacy, and obtaining adequate consent during a remote prescribing consultation must be embedded in practice. This practice aligns with professional regulations and establishes trust, and optimises treatment adherence, thereby reducing the disease burden (Scottish Government 2023; Royal Pharmaceutical Society 2021). Challenges to ensuring confidentiality arise in remote consultation, especially when other people are present, resulting in guarded conversations and non‐disclosure of clinically relevant information. Full disclosure is vital for accurate prescribing decisions, and an incomplete patient history can result in under‐ or over‐prescribing, missed warning signs, and increased harm (Rosen et al. 2022; Wherton et al. 2020).

For informed consent to be valid, the patient must be informed and have the capacity to understand the fundamentals of remote prescribing, including alternative consultation methods (GMC 2019; HCPC 2019; NMC 2019). Without this clarity, the patient's ability to provide informed consent may be compromised (Cave and Law 2019). Therefore, effective communication before, during, and after a remote prescribing consultation is crucial. However, the reviewed literature lacks clarity regarding practitioner preparation, education, and support to effectively conduct remote prescribing consultations. Inadequate training in remote prescribing poses challenges, potentially leading to unnecessary in‐person consultations or inadequate decision‐making (Chaudhry et al. 2020; Dixon et al. 2022). Future policy and practice should consider how remote consultation is embedded within pre‐ and post‐qualifying prescribing and consultation education and the impact this education has on remote prescribing and safety. As the UK leads in non‐medical prescribing practice, developing policy practice and research in this could have global reach and potentially improve medicines safety worldwide.

The critical importance of access to patient records during remote consultations is evident from the review. Comprehensive patient information enables clinicians to make more informed decisions, identify differential diagnoses, and address clinical risks (Atherton et al. 2018; Dixon et al. 2022; King et al. 2024). Conversely, limited, inaccurate, or patient‐reported information (Uscher‐Pines et al. 2020) increases clinical risk and is a frequent factor associated with prescribing errors (Hodkinson et al. 2020). A possible solution would be for patients to retain access to their own medical records, which could be shared before or during a remote prescribing consultation.

This review highlights strategies that can support remote assessment, such as taking photographs, self‐monitoring and self‐examination. These help to mitigate inappropriate prescribing or poor management of existing conditions. Research understanding of how practitioners' approach remote consultations when patient records are not available would be beneficial. This would identify best practice and innovative solutions for hard‐to‐reach populations with limited access to medicines.

Application of prescribing guidance to support decision making in remote consultation underpins safety and governance. There is evident alignment with the high‐level principles and regulatory guidance with regard to documentation, rationale for decisions, and safety netting when prescribing remotely. It is also clear that escalation to face‐to‐face consultations was deemed necessary in ‘red flag’ or emergency situations. This provides valuable insight into managing risk for novice remote non‐medical prescribers and would be an essential component of education in this area.

Several studies examined in this review emphasised the need to establish robust systems and infrastructure to support safe, remote prescribing. However, these elements do not form part of the high‐level principles. The wider literature underscores the importance of adequate training, clinical risk assessments, policies, and sufficient time for decision making as critical aspects for effective implementation (Tyrer et al. 2022). In addition, interoperable IT systems, standardised processes, and cross‐sector communication are all integral to safe remote prescribing, yet their absence from high‐level principles is notable. Mismatching in electronic systems and lack of shared care records can lead to medication errors and ultimately greater risks for safe prescribing (Shamsuddin et al. 2021). We recommend a more in‐depth exploration of how infrastructure‐related issues can be more thoroughly addressed and integrated into future iterations of high‐level principles.

## 5 Strengths and Limitations

The systematic methodology employed in this review enhances its rigour and clarity. However, most of the studies were conducted during the COVID‐19 pandemic and may not reflect the current breadth of practice in this area. Finally, the high‐level principles are UK‐focused and only a small number of studies were conducted in the UK. Therefore, conclusions about how the high‐level principles are applied in remote prescribing may not be generalisable.

## 6 Conclusion

This review highlights the complexities of consultation and governance in remote prescribing. It has showcased the innovative strategies that practitioners use to overcome these challenges but has also highlighted significant gaps, such as how non‐medical prescribers in the United Kingdom approach remote prescribing. The review underscores the need to understand how policy and governance are being embedded in remote prescribing practice and raises important questions about what happens when these resources are not available.

## Author Contributions

Made substantial contributions to conception and design, or acquisition of data, or analysis and interpretation of data; M.L., S.R., R.E.P., S.M. Involved in drafting the manuscript or revising it critically for important intellectual content; M.L., S.R., R.E.P., S.M. Given final approval of the version to be published. Each author should have participated sufficiently in the work to take public responsibility for appropriate portions of the content; M.L., S.R., R.E.P., S.M. Agreed to be accountable for all aspects of the work in ensuring that questions related to the accuracy or integrity of any part of the work are appropriately investigated and resolved. M.L., S.R., R.E.P., S.M.

## Conflicts of Interest

The authors declare no conflicts of interest.

## Supporting information


**Data S1:** Example of search terms.

**Data S2:** Eligibility criteria rationale.

## Data Availability

The data that support the findings of this study are openly available.
