# Supplementary material for: Adherence to Remote Prescribing Principles by Medical and Non‐Medical Prescribers; a Scoping Review
Source: J Adv Nurs. 2025 Sep 8;82(5):4610–24. doi: 10.1111/jan.70198 (PMC13069193; doi:10.1111/jan.70198)
Supplement: Supplementary file 1 — Data S1: Example of search terms. [file JAN-82-4610-s002.docx]

Supplementary File 1

***Example Search Terms***

| Search Term | Alternative term |
| --- | --- |
| Governance structures | Governance OR Safety OR Safe OR Guidance OR Guidelines OR clinical guides OR Policy OR authority OR control OR quality OR “quality assurance” OR Principles OR Adequacy |
| Remote Consultations | Remote Consultation OR e-consultations OR telephone consultations OR digital healthcare OR Remote diagnosis OR virtual consultations OR Video consultations or online consultations Remote Consultation OR e-consultations OR telephone consultations OR digital healthcare OR Remote diagnosis OR virtual consultations OR Video consultations or online consultations |
| Remote Prescribing | Remote prescription* OR e-prescription* OR telephone prescribing OR e-prescribing OR Video prescribing OR digital prescribing OR online prescribing OR virtual prescribing |
