# Supplementary material for: Adherence to Remote Prescribing Principles by Medical and Non‐Medical Prescribers; a Scoping Review
Source: J Adv Nurs. 2025 Sep 8;82(5):4610–24. doi: 10.1111/jan.70198 (PMC13069193; doi:10.1111/jan.70198)
Supplement: Supplementary file 2 — Data S2: Eligibility criteria rationale. [file JAN-82-4610-s001.docx]

**Supplementary File 2.**

Eligibility Criteria Rationale

1. The criteria included papers that addressed the scoping review questions.
2. As per criterion 1.
3. Criteria were of all health care settings worldwide
4. Describes remote prescribing platforms – terms were developed and agreed upon with the subject librarian (McGowan et al, 2016)

Only papers published from 2007 onwards were included. After an extensive literature search, it was identified that the legal implementation of electronic prescribing – an essential enabler of remote prescribing began in the mid-2000s. In 2007, the United Kingdom was among the first countries to introduce a legal framework supporting e-prescribing (Skelton, 2015).

Only papers published in English were included, as the research team did not have a budget for translation services.

**Reference list**

McGowen J., Sampdon M., Salzwedel D., Cogo E., Forester V and Lefebvre (2016) PRESS Peer Review of Electronic Strategies: 2015 Guideline Statement. J clin Epidemoil. Jul;75:40-6.

Skelton, T. (2015). 10 years of interoperability in action: A historical look at e-prescribing. Surescripts. <https://surescripts.com/news-center/intelligence-in-action/access-security-and-performance/10-years-of-interoperability-in-action-a-historical-look-at-e-prescribing>
